# Vesicular Release and Uptake of Circular LSD1-RNAs from Non-Cancer and Cancer Lung Cells

**DOI:** 10.3390/ijms241813981

**Published:** 2023-09-12

**Authors:** Joelle Noriko Galang, Yefeng Shen, Ulrike Koitzsch, Xiaojie Yu, Hannah Eischeid-Scholz, Daniel Bachurski, Tilman T. Rau, Christina Neppl, Marco Herling, Bianca Bulimaga, Elena Vasyutina, Michal R. Schweiger, Reinhard Büttner, Margarete Odenthal, Maria M. Anokhina

**Affiliations:** 1 Institute of Pathology, University Hospital of Cologne, 50937 Cologne, Germany; joellenoriko.galang@ru.nl (J.N.G.); yefeng.shen@uk-koeln.de (Y.S.); xiaojie.yu@uk-koeln.de (X.Y.); hannah.eischeid-scholz@uk-koeln.de (H.E.-S.); mbulimag@smail.uni-koeln.de (B.B.); reinhard.buettner@uk-koeln.de (R.B.); 2 Center for Molecular Medicine Cologne, University of Cologne, 50937 Cologne, Germany; mschweig@uni-koeln.de; 3 CECAD Center of Excellence on Cellular Stress Responses in Aging-Associated Diseases, University of Cologne, 50937 Cologne, Germany; daniel.bachurski@uk-koeln.de; 4 Department I of Internal Medicine, University Hospital of Cologne, 50937 Cologne, Germany; marco.herling@medizin.uni-leipzig.de (M.H.);; 5 Institute of Pathology, University Hospital of Duesseldorf, 40225 Duesseldorf, Germany; Tilman.Rau@med.uni-duesseldorf.de (T.T.R.); Christina.Neppl@med.uni-duesseldorf.de (C.N.); 6 Department of Hematology, Cellular Therapy and Hemostaseology, University of Leipzig, 04103 Leipzig, Germany; 7 Institute for Epigenetics, University Hospital of Cologne, 50937 Cologne, Germany

**Keywords:** circular RNA, extracellular vesicles, KDM1a, lung cancer

## Abstract

Lysine-specific demethylase 1 (LSD1) is highly expressed in many cancer types and strongly associated with cancer progression and metastasis. Circular RNAs (circRNAs) are produced by back-splicing and influence the interactive RNA network by microRNA and protein sponging. In the present study, we aimedto identify circRNAs that derive from the LSD1-encoding *KDM1A* gene, and to investigate their potential to be released and uptaken by lung cancer versus non-cancer epithelial cells. We identified four circLSD1-RNAs by RT-PCR with divergent primers, followed by sequencing. The expression level of circLSD1-RNAs was then studied by quantitative PCR on cellular and extracellular fractions of lung cancer (PC9) and non-cancer primary small airway epithelial (PSAE) cells. Moreover, we established the transgenic overexpression of circLSD1-RNAs. We show that circLSD1-RNAs are primarily located in the cytoplasm, but are packaged and released from lung cancer and non-cancer cells by extracellular vesicles (EVs) and ribonucleoprotein (RNP) complexes, respectively. Proteomics demonstrated a different protein pattern of EV fractions released from PC9 versus PSAE cells. Importantly, released circLSD1-RNAs were differently taken up by PSAE and PC9 cells. In conclusion, our findings provide primary evidence that circLSD1-RNAs participate in the intercellular communication of lung cancer cells with the tumor environment.

## 1. Introduction

Lysine-specific histone demethylase 1 (LSD1) is a histone-modifying enzyme encoded by the *KDM1A* gene. Depending on its binding partner, LSD1 mediates alterations that cause transcription activation or repression [1,2]. LSD1 is highly expressed in a variety of cancer types, including lung cancer, and is significantly associated with poor prognosis [3,4]. Lung cancer is one of the most prevalent cancer types and the leading cause of cancer-related deaths worldwide [5]. It is classified into small-cell lung cancer and non-small-cell lung cancer (NSCLC). Among them, lung adenocarcinoma (LUAD) is an NSCLC form, which is the most common lung cancer subtype [6]. Due to its poor 5-year survival rate, novel therapeutic strategies are needed [7]. Therefore, recent research has focused on cellular and molecular characterization, including the identification of genetic and epigenetic alterations, that may contribute to its development [8,9].

In addition, over the last decade, the understanding of intercellular communication pathways between the tumor and the tumor microenvironment (TME) during cancer progression, resistance, and therapy escape has been brought onto center stage. This considers the effect of released soluble signaling molecules such as cytokines, growth factors, and neurotransmitters on downstream signaling cascades, which dictates cell behavior [10,11]. Apart from these soluble factors, the transferred DNA and RNA cargo molecules are shown to be involved in intercellular communication [12]. There are several types of RNA transcripts with various functions in protein synthesis, transcription regulation, and interference [13], which are suggested to be released from cells and be involved in cellular crosstalk [14].

Circular RNAs (circRNAs) are single-stranded closed-loop RNAs, formed through a back-splicing mechanism, where the 5′ and 3′ end of pre-mRNAs are covalently bound [15]. This closed-loop structure results in greater stability and resistance to the RNase degradation of circRNAs compared to other RNA structures [16]. Currently, there are three widely accepted models for circRNA biogenesis: (1) RNA-binding protein mediated circularization [17], (2) intron pairing-driven circularization [18], and (3) lariat-driven circularization [19]. These back-splicing mechanisms result in various circRNA types containing exons, introns, or a combination of both. CircRNAs function as miRNA sponges, regulators of gene transcription, and vehicles for RNA-binding proteins, while other circRNAs may be translated into functional proteins [20]. Back-splicing was thought to compete with the splicing of pre-mRNA into mature mRNA, since circRNAs are generated from the same pre-mRNA. Thus, the production of circRNA potentially contributes to the regulation of its corresponding mRNA expression level [21].

The advent of next-generation sequencing enabled the sequencing of circRNAs throughout the genome, resulting in the detection of numerous circRNAs derived from virtually all mammalian genes [22,23]. However, most of these circRNAs have not been functionally characterized. Some, whose functions have been investigated, were shown to play important roles in disease progression, including carcinogenesis [24].

Moreover, circRNAs were not only detected in tissues but also as extracellular RNAs in the blood stream, and different patterns of circulating circRNAs have been described in serum of cancer patients versus healthy donors [25]. However, how circRNAs are released and if they are transferred from one cell to another, and thereby participating in intercellular signaling or contributing to disease spread or inhibition, is not well understood. The release and transfer of non-coding RNAs such as microRNAs is usually facilitated by extracellular vesicles or ribonucleoprotein complexes [26,27]. Extracellular vesicles (EVs) are lipid-enclosed vesicles released by the cell. The lipid membrane protects the vesicle’s contents from degradation in the extracellular space [28]. They are classified according to their biogenesis and size. Some EVs, called microvesicles or ectosomes, are formed from the budding of the plasma membrane [29]. Other EVs are called exosomes, which derive from the endosome and measure 30–150 nm in diameter. Theyare formed by the invagination of the endosome, forming multivesicular bodies (MVBs) containing intraluminal vesicles (ILVs). MVBs fuse to the plasma membrane and the ILVs are released as exosomes [30]. EVs contain various types of molecular cargo including DNAs, mRNAs, noncoding RNAs, and small proteins, depending on the cell of origin [25]. Although exosome formation was originally identified as a process that mainly facilitates waste removal by trafficking to the lysosome for degradation, it has been shown that exosomes may be taken up by other cells through ligand binding or plasma membrane fusion [31]. This underlines the potential for EVs to participate in intercellular communication in the tumor microenvironment.

In the present study, we focused on the release and uptake of circRNAs deriving from the LSD1 gene *KDM1A*. Numerous circLSD1-RNAs were computationally predicted or identified by bulk circRNA sequencing studies. These circLSD1-RNAs are listed in the circRNA database (circbase.org, accessed on 12 March 2023), but only hsa_circ_0009061 has been characterized to date [32].The *KDM1A* gene consists of 21 exons with alternative exons 2a and 8a, resulting in additional LSD1 protein isoforms. The alternative inclusion of exon 8a is observed only in neuronal cells, whereas the alternative inclusion of exon 2a occurs ubiquitously. Since circRNA biogenesis is often associated with alternative splicing processes, we studied circLSD1-RNAs from the *KDM1A* exons 2, 2a, and 3 locus, and identified and characterized four circRNAs. They were shown to be present in both PC9 LUAD cells and primary small airway epithelial (PSAE) cells in varying expression levels, reflecting also the expression level of LSD1 mRNA. Moreover, we monitored whether these circLSD1-RNAs are released by donor cells and subsequently taken up by acceptor cells, and if this genetic transfer occurs with the aid of small EVsor if RNA binding proteins are also associated. Importantly, we proved the differences in the release and uptake of circLSD1-RNA between lung cancer and non-cancer lung epithelial cells. Looking into the mechanism of the circLSD1-RNA transfer and its differences in cancer and non-cancer cells, our findings indicate the contribution of circRNAs in the intercellular communication between cancer and non-cancer cells.

## 2. Results

### 2.1. Four circRNAs Are Derived from Exons 2, 2a, and 3 of the LSD1 Parental Gene KDM1A

**Figure 1:**
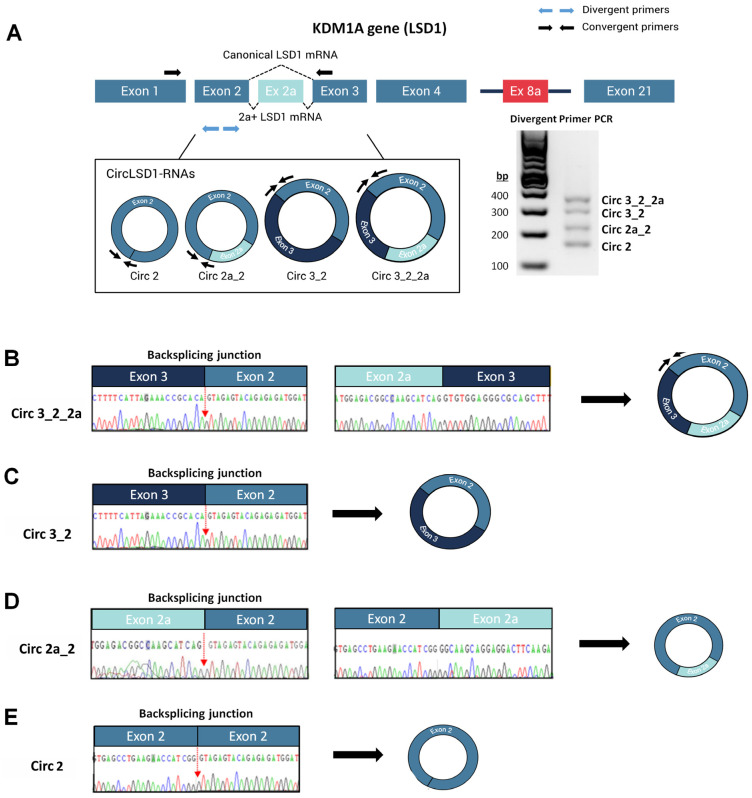
Identification and characterization of circLSD1-RNAs in PC9 LUAD cells. (**A**) Schematic diagram of the *KDM1A* gene locus, including 21 exons with alternative exons 2a and 8a. Location of the divergent (blue arrows) and convergent primers (black arrows) used to identify the circLSD1-RNAs and LSD1 mRNA isoforms, respectively, are indicated. The agarose gel image shows the four circLSD1-RNAs identified. (**B**–**E**) Representative images of back-splicing junctions, identifying (**B**) circ 3_2_2a, (**C**) circ 3_2, (**D**) circ 2a_2, and (**E**) circ 2 LSD1 RNAs by Sanger sequencing.

To study the circRNA transcripts from the LSD1-encoding gene locus, we used convergent and divergent sets of primers targeting the *KDM1A* exonic sequences of exon 2, 2a, and 3 in reverse-transcription-PCR amplification assays (Figure 1A). In addition to the linear alternatively spliced LSD1 transcript isoforms, either skipping or including exon 2a of the *KDM1A* gene, we found potential circLSD1-RNAs by using divergent primer sets. The four amplicons, ranging in size from 160 to 360 bp, were then purified and sequenced (Figure 1A). Pursuant to the back-splicing junctions shown by sequencing, we identified four circLSD1-RNAs harboring either exon 3, exon 2, and exon 2a (Figure 1B); exon 2 and 3 (Figure 1C); exon 2 and 2a (Figure 1D); or only exon 2 (Figure 1E), and named them accordingly: circ 3_2_2a, circ 3_2, circ 2a_2, and circ 2.

The circRNAs carrying exons 3, 2, and 2a, as well as the circRNA that includes the exons 2a and 2, were annotated as circ_0009061 and circ_0112434, respectively, whereas circ 3_2 and circ 2 were not yet described (Table 1).

### 2.2. CircLSD1-RNAs Are Mainly Localized in the Cytoplasm

**Figure 2:**
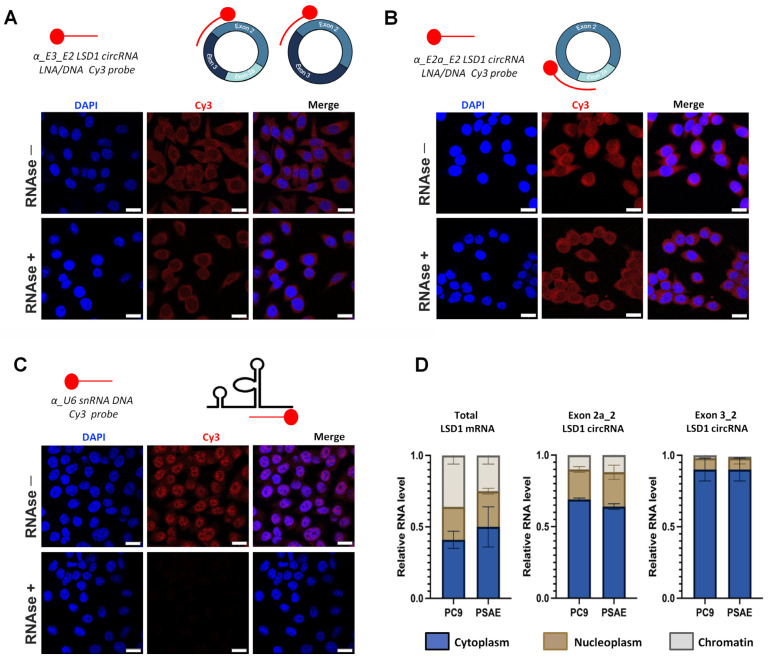
Localization of circLSD1-RNAs. (**A**,**B**) Fluorescence in situ hybridization in PC9 cells using antisense LNA/DNA probes fluorescently tagged with Cy3 to monitor presence of circLSD1-RNAs: (**A**) circ 3_2 and circ 3_2_2a; (**B**) circ 2a_2 in the PC9 cells before and after RNase R treatment. (**C**) Fluorescence in situ hybridization in PC9 cells using an antisense DNA probe fluorescently tagged with Cy3 to visualize U6 snRNA as a positive control, showing efficient nuclear probe penetration as well as RNase R digestion. The scale bar of FISH images of subfigures (**A**–**C**) represents 20 µm. (**D**) qPCR showing relative RNA levels of total linear LSD1 mRNA, and exon 2a_2 and exon 3_2 LSD1 circRNAs in the cytoplasm, nucleoplasm, and chromatin fractions of PC9 and PSAE cells (*n* = 3).

Next, we studied the localization of the circLSD1-RNAs, using probes that target either the exon2a_exon2 back-splicing junction of circ 2a_2, or the exon3_exon2 junction of circ 3_2_2a and circ 3_2, and performed fluorescence in situ hybridization (FISH) on the PC9 LUAD cells. Whereas the scrambled probe, used as a negative control, showed no signal (Supplemental Figure S1A), pronounced signals were observed mainly in the cytoplasm and, to a lower extent, in the nuclei of PC9 cells using the circLSD1-RNA-specific probes (Figure 2A,B).

To prove that the moderate signals in the nuclei are not due to the low nuclear penetration efficiency of the probes, we used a probe which targets the U6 small nuclear (sn) RNA. U6-FISH showed marked nuclear hybridization signals, confirming no technical reasons for the low nuclear circLSD1-RNA signals (Figure 2C).

CircRNAs are resistant to RNAase R degradation. To validate that the designed circLSD1-RNA probes recognized the exon junctions of circRNA, we treated the cell specimens with RNase R prior to hybridization. A separate setup of untreated PC9 cells served as controls. Despite RNase R treatments, the probe signals were unchanged in the PC9 cells hybridized with the circLSD1-RNA antisense probes (Figure 2A,B), whereas, after the RNase R degradation of linear RNAs in PC9 cells hybridized with the α-U6 snRNA probe, no fluorescent signals were observed (Figure 2C).

To confirm the predominant localization of circLSD1-RNAs in the cytoplasm, we prepared cytoplasm, nucleoplasm, and chromatin fractions of cancer and non-cancer cells (Supplemental Figure S1B) and quantified the total LSD1 mRNA and the circLSD1-RNAs. To this end, on the one hand, primers targeting exons 1 and 2 were used, allowing the quantification of the total LSD1 mRNA expression. On the other hand, the quantification of circLSD1-RNAs was performed by qPCR with convergent primer sets targeting specific back-splicing junctions of the respective circLSD1-RNAs (Supplemental Table S1). In contrast to linear LSD1 mRNA, most circLSD1-RNAs were found in the cytoplasm as we had also shown by FISH. Furthermore, these studies revealed that circLSD1-RNA was equally distributed in the compartments of LUAD PC9 cells and in non-cancer primary small airway epithelial (PSAE) cells (Figure 2D).

### 2.3. CircLSD1-RNAs Are Differentially Expressed in PSAE Non-Cancer and PC9 Cancer Cells and Are Found as Extracellular RNA, Circulating in the Blood 

**Figure 3:**
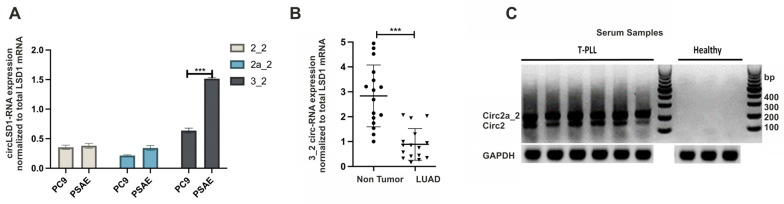
CircLSD1-RNAs in LUAD versus non-cancer lung cells and in serum samples of T-PLL patients and healthy donors (**A**) CircLSD-RNA-specific quantitative PCR (qPCR) targeting circ 2_2, circ 2a_2, and circ 3_2 LSD1 RNA in PC9 LUAD cells in comparison to lung epithelial PSAE cells. Significance *p* < 0.001 is indicated by ***. (**B**) Circ 3_2 LSD1 RNA levels, determined by qPCR in LUAD (*n* = 16) and in the matching non-tumor areas. (**C**) PCR amplification with divergent primers for circLSD1-RNAs of serum samples of T-PLL patients (left) and healthy donors (right), followed by submarin agarose gel electrophoresis.

Further analysis of the circLSD1-RNA expression revealed that, in comparison to PC9 cancer cells, PSAE cells have a higher proportion of circLSD1-RNA production relative to the total LSD1 mRNA expression (Figure 3A). Notably, the circLSD1-RNAs with the exon3_exon2 back-splicing junction are the most downregulated circLSD1-RNAs in cancer PC9 versus non-cancer PSAE cells (Figure 3A).

Therefore, we next extracted RNA from 16 LUAD samples and the matching adjacent non-tumor tissues, and quantified the expression levels of circ 3_2 LSD1-RNAs by reverse transcription, followed by quantitative real-time PCR (qPCR). Consistent with our observations in cancer PC9 and non-cancer PSAE cells, circ 3_2 LSD1-RNAs were significantly fewer in the analyzed LUAD tissues in comparison to the matching non-tumor areas (Figure 3B).

The decrease of miRNAs in cancer tissues is often based on their release into the cell environment or the blood stream [33]. In order to address the question of if circLSD1-RNAs also occur as extracellular RNAs and if they are released into the blood, we studied the expression levels in serum samples. Although, from LUAD patients, no serum samples were available, we were able to investigate circLSD1-RNAs in serum samples from patients with T-cell prolymphocytic leukemia (T-PLL) versus healthy controls. Indeed, in the T-PLL serum samples, we found high levels of circ 2 and 2a_2 LSD1-RNAs, whereas, in samples of healthy donors, no circLSD1-RNAs were detected (Figure 3C). This indicates that circLSD1-RNAs can be released from the leukemia cells to the blood stream.

### 2.4. CircLSD1-RNAs Are Differently Released by LUAD PC9 and Non-Cancer PSAE Cells

**Figure 4:**
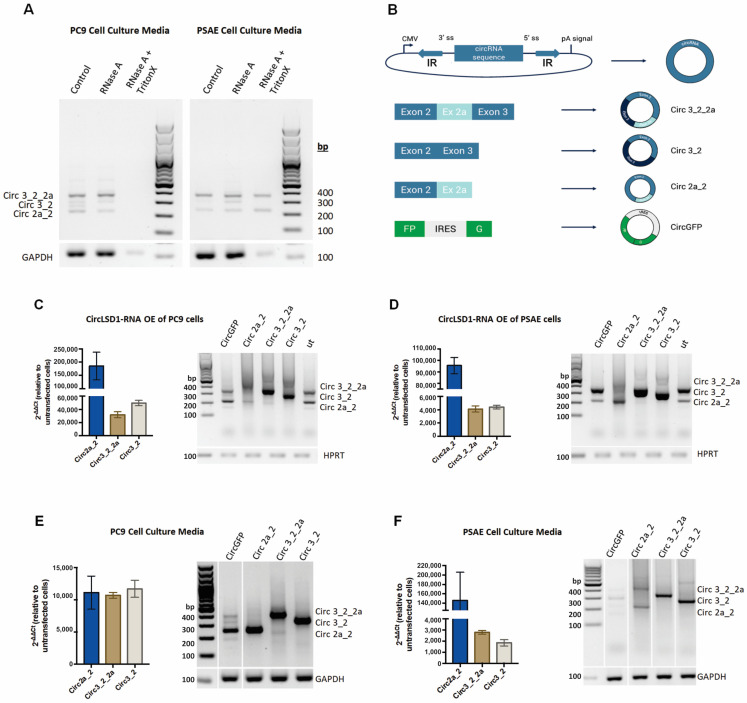
Release of circLSD1-RNAs in cell culture medium of PC9 and PSAE. (**A**) PCR amplification with divergent primers for circLSD1-RNAs of cell culture media from PC9 (left) and PSAE (right) cells treated with RNase A or a combination of RNase A and Triton X-100. (**B**) Schematic diagram of circLSD1-RNA plasmids for overexpression. Inverted complementary (IR) sequences flanked the circRNA sequence of interest, forcing back-splicing in the cell. Overexpression plasmids were prepared for circ 3_2_2a, circ 3_2, circ 2a_2, and circGFP, which serve as a control to monitor occurrence of back-splicing. (**C**,**D**) CircLSD1-RNA overexpression was proven in PC9 (**C**) and PSAE cells (**D**) by means of qPCR using convergent primers for each circLSD1-RNA (circ 2a_2, circ 3_2_2a, and circ 3_2) (shown as bar graphs, left) or qualitative PCR with divergent primers, recognizing all three overexpressed circLSD1-RNAs (right). (**E**,**F**) circLSD1-RNAs released into cell culture medium upon transgenic overexpression in PC9 cells (**E**) and PSAE cells (**F**) were shown after qPCR using the convergent primer sets (shown as bar graphs, left). The fold changes of circLSD1-RNAs were calculated by the ΔΔCt method, using the levels of circLSD1-RNAs obtained from cell culture supernatants of untransfected cells as references. Agarose gel image after PCR amplification with divergent primers for circLSD1-RNAs are also shown (right).

Since circLSD1-RNAs were found to be differently expressed in cancer and non-cancer cells and appeared as extracellular RNA in leukemia patient serum samples, we examined their release from PC9 cancer and PSAE non-cancer cells. The qualitative PCR analysis using the divergent primer sets clearly showed the extracellular presence of circ 3_2_2a, circ 3_2, and circ 2a_2 circLSD1-RNAs in the supernatants of PC9 and PSAE (Figure 4A).

It has also been previously shown that extracellular miRNAs and extracellular circRNAs are often aggregated with proteins and packaged into vesicles, and, thereby, are protected from degradation [34]. To study, if the extracellular circLSD1-RNAs are exported by vesicles, we collected cell culture media from PC9 and PSAE cells and treated them with the RNase A endonuclease. Qualitative and quantitative RT-PCR analyses were then performed using divergent primers to amplify circLSD1-RNAs or with convergent primer sets, recognizing the back-splicing junctions, by means of qPCR. The resistance of extracellular circLSD1 RNAs, deriving from both PC9 and PSAE cells, to RNAse A degradation showed efficient protection. However, simultaneous treatment with the non-ionic detergent Triton X-100, solubilizing membranes [35], significantly restored the RNAse A efficiency in supernatants from PC9, but not from PSAE cells (Figure 4A). The resistance of circLSD1-RNAs that are released from PSAE cells to RNAseA degradation even despite Triton X-100 treatment indicates an additional protection of extracellular circLSD1-RNA released from non-cancer PSAE cells by RNA binding proteins, assembled in ribonucleoprotein (RNP) complexes.

To study the release of circLSD1-RNAs and follow individual circLSD1-RNAs, we used the vector construct, developed by Jeremy Wilusz’s group [36]. Using these plasmid constructs, circularization is induced by inverted repeats (IR) flanking the circularized exon, which presumably brings the splice acceptor (SA) and splice donor (SD) sites into close proximity for back-splicing [37]. Thus, RNA transcribed from this vector system efficiently circularizes due to inverted repeats from ZSCAN1 pre-mRNA, flanking the exons of choice and forming the hairpin-loop. We cloned the LSD1 exon sequences of the circLSD1-RNAs, deriving from the exon 2-2a-3 *KDM1A* locus, and prepared the plasmids with expression cassettes of circ 3_2_2a, circ 3_2, and circ 2a_2 (Figure 4B). As a control, a circGFP plasmid was designed, in which the GFP is produced only when the circRNA is formed (Figure 4B, Supplemental Figure S2A). Around 90% of the control cells that have been transfected with the circGFP-expressing plasmid showed a fluorescent signal 24 h post-transfection. The translation ability of circGFP-RNA demonstrated the efficiency of back-splicing from the constructed plasmids and correct circRNA production (Supplemental Figure S2B).

Using qualitative and quantitative PCR, we showed that all circLSD1-RNAs of the exons 2, 2a, and 3 locus were efficiently overexpressed (Figure 4C,D). Cell supernatants were collected 24 or 48 h post-transfection of PC9 and PSAE cells, to determine circLSD1-RNA levels in the cell culture media. The presence of GAPDH mRNA, which is also loaded into small EVs, was used as a housekeeping control. The amounts of GAPDH mRNA, released by both PC9 and PSAE cells into the cell supernatants, were not changed after circRNA overexpression. Overexpressed circRNAs were efficiently released by both PC9 and PSAE cells (Figure 4E,F). Corresponding to the findings on endogenous extracellular circLSD1-RNAs, the RNase A treatment alone did not degrade the released circLSD1-RNAs circ 3_2_2a, circ 3_2, and circ 2a_2, as well as circGFP, after their overexpression in PC9 cells. However, the simultaneous treatment of RNase A plus Triton X-100 led to a distinct degradation of extracellular circLSD1-RNA and circGFP levels in PC9 cell supernatants (Supplemental Figure S2A,B). Moreover, similarly to the results on endogenously released PSAE circLSD1-RNAs, we show that the extracellular circLSD1-RNAs deriving from PSAE non-cancer cells after transgenic overexpression is resistant to RNAse A degradation even when Triton X membrane solubilization was used (Supplemental Figure S3A,B).

Therefore, we assume that, in agreement with the endogenous circLSD1-RNAs released from PSAE non-cancer cells, the transgenically expressed circLSD1-RNAs are also associated to RNA-binding proteins and released as RNP complexes.

### 2.5. Extracellular Vesicles and RNP Complexes, Transporting circLSD1-RNAs

**Figure 5:**
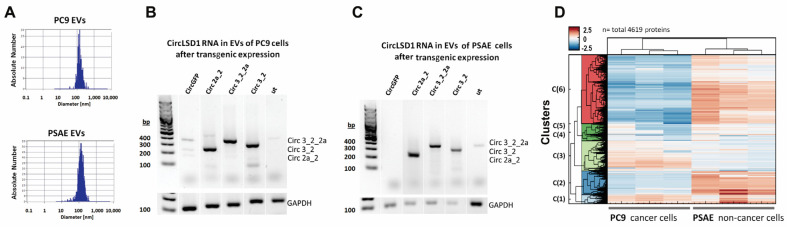
EVs and RNP aggregates, released from PC9 and PSAE cells. (**A**) Size distribution of isolated EVs from PC9 and PSAE cells shown by nanoparticle tracking analysis. (**B**,**C**) Representative agarose gel images showing the amplicons using divergent primer sets on circLSD1-RNAs isolated from EVs which were released from PC9 (**B**) and PSAE cells (**C**) after the respective overexpression. (**D**) Protein pattern of the EV fraction obtained from PC9 and PSAE cells after mass spectrometry and hierarchical cluster analysis, highlighting six clusters (C1–6).

Next, we characterized the extracellular vesicles and RNP complexes that are released by PC9 and PSAE cells and that transport the circLSD1-RNAs to the extracellular space. To this end, we isolated the vesicular fractions with EVs and RNP complexes by the precipitation method. First, we determined the size of the EVs released from PC9 and PSAE cells by nanoparticle tracking analysis (NTA) technology. The size of EVs from both PC9 and PSAE cells were around 100 nm in diameter (Figure 5A), similar to the size of exosomes/small EVs, being generally around 40–120 nm in diameter [38,39]. After the overexpression of circLSD1 RNAs in PC9 and PSAE cells, high amounts of the respective overexpressed circLSD1-RNAs were found in the extracellular vesicular fraction (Figure 5B,C). Importantly, after transgenic expression in PC9 and PSAE cells, we also observed EV sizes of around 100 nm in diameter (Supplemental Figure S4A,B).

**Figure 6:**
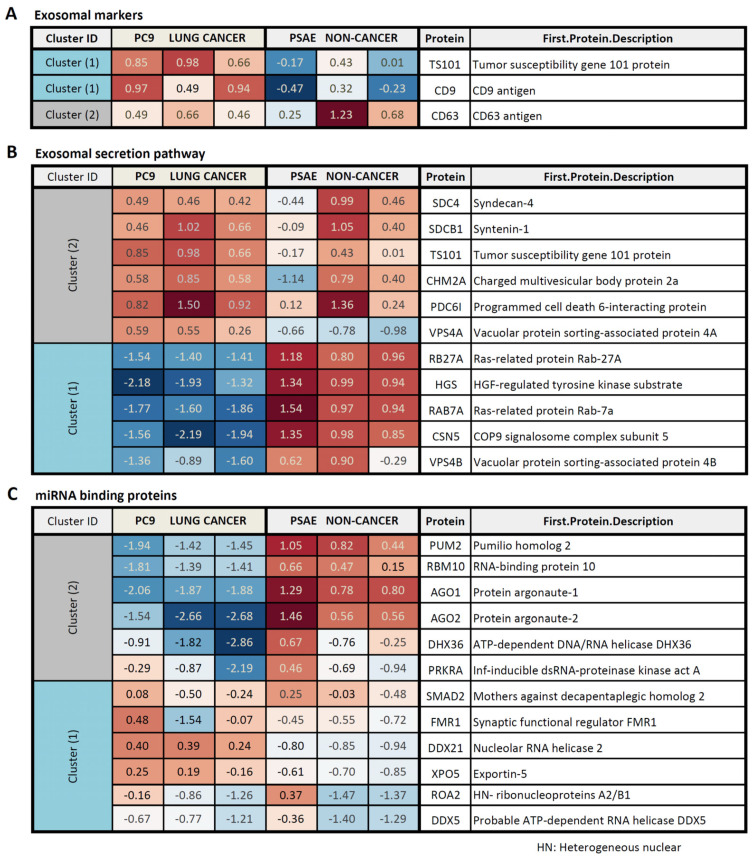
Protein pattern of EV fractions, obtained from PC9 and PSAE cells. Different pattern of EV marker proteins (**A**), proteins involved in the EV secretory pathways (**B**), and miRNA-binding proteins (**C**) in PSAE non-cancer versus PC9 cancer cells. The fold change (FC) between the levels in EV fractions, obtained from PC9 versus PSAE cells, is indicated.

Additionally, by Western blot, we identified the EV markers TSG101, CD63, and CD9 in the extracellular vesicular fraction obtained from PC9 cells, but to a much lesser extent, barely detectable in the fraction from PSAE cells (Supplemental Figure S4C,D). In agreement with the Triton X resistance and protection of the circLSD1-RNAs released by PSAE cells, these findings provide further evidence for the differences in the vesicular RNA packaging and protein aggregation in PSAE versus PC9 cells. Therefore, we studied the protein composition of the EV fractions from PC9 and PSAE cells by proteomic analysis using mass spectrometry. As shown in Figure 5D, 4619 proteins were detected and a hierarchical cluster analysis revealed pronounced differences in six clusters (clusters 1–6). After examining the data in more detail, we found that there was a substantial difference in the abundance of EV markers and peptides associated with EV trafficking in the EV fractions of PC9 and PSAE cells (Figure 6A,B). The low EV formation and low occurrence of EV marker proteins by PSAE cells confirmed our previous immunoblotting data shown in Supplemental Figure S4C,D. Furthermore, the peptide pattern of EV fractions from PSAE cells differed in RNA/miRNA-binding proteins as shown in Figure 6C.

Further pathway analysis revealed that mainly the proteins contributing to EV RNA processing, including decay and modification processes, differed in the EV fractions, which we obtained from PC9 and PSAE cells (Table 2). Detailed analysis of the RNA processing proteins proved the pronounced difference in proteins, which are involved in the RNA binding, sensing, and decay mechanisms, between the EV fractions of PC9 and PSAE cells (Supplemental Figure S5A,B).

### 2.6. CircLSD1-RNAs Are Differentially Taken Up by PSAE and PC9 Cells

**Figure 7:**
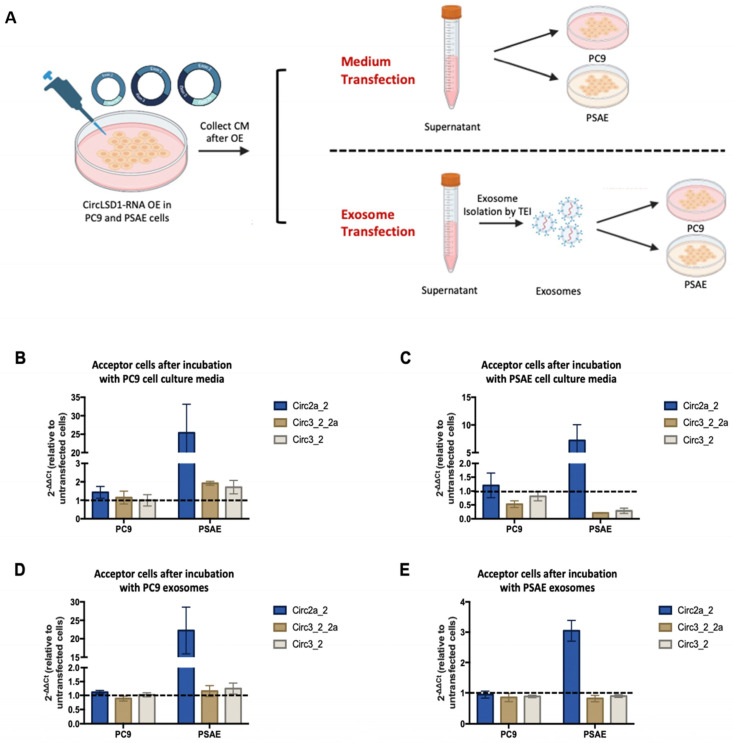
Released circLSD1-RNAs can be taken up by PC9 and PSAE acceptor cells via extracellular vesicles. (**A**) Schematic diagram of experiments performed to monitor circLSD1-RNA uptake in PC9 and PSAE cells. After circLSD1-RNA overexpression in donor cells, cell culture media are collected and directly used to culture acceptor cells in the medium transfection setups. On the other hand, small EV transfection setups have an intermediate step where EVs are isolated, before being added to the acceptor cells. (**B**,**C**) CircLSD1-RNA levels in PC9 and PSAE acceptor cells after incubation with cell culture medium from (**B**) PC9 and (**C**) PSAE cell donors after circLSD1-RNA overexpression (*n* = 3). Fold change is relative to acceptor cells receiving cell culture medium from untransfected cells with HPRT as the housekeeping gene. (**D**,**E**) Fold change in uptake of circLSD1-RNA in PC9 and PSAE acceptor cells after transfection with EVs from (**D**) PC9 and (**E**) PSAE donor cells after circLSD1-RNA overexpression (*n* = 3).

Next, we studied the potential for extracellular circRNA uptake by the PC9 cancer and PSAE non-cancer cells. Two approaches were designed, in which we treated both cell types either with conditioned media or with the isolated EVs from PC9 or PSAE, that transgenically expressed the circLSD1-RNAs (Figure 7A).

Hence, we first induced circLSD1-RNA overexpression in PSAE or PC9 donor cells by plasmid transfection as described previously. The following day, we collected the cell culture media and used the conditioned media to culture newly seeded PSAE or PC9 acceptor cells. We called this approach “media transfection”. In the other approach, which we called “ small EV transfection”, the collected conditioned media were further processed to isolate the EVs. The purified EVs were then added to the PSAE and PC9 acceptor cells.

CircLSD1-RNA levels in both PC9 and PSAE acceptor cells were then determined by qPCR. In comparison to acceptor cells receiving media from untransfected, non-overexpressing cells (negative control) or from donor cells after circGFP overexpression (mock control), there was a notable increase in circLSD1-RNAs in acceptor cells after both media and small EV transfection (Figure 7A–D). This effect was seen in both medium and EV transfection, but the uptake appears to be higher in acceptor cells receiving the conditioned medium of EVs from PC9 donor cells than the uptake of the conditional medium or EV originating from PSAE donor cells (Figure 7A–D).

Moreover, PSAE acceptor cells showed a higher uptake of circLSD1-RNAs compared to PC9 acceptor cells. Among the different circLSD1-RNAs, circ 2a_2 is most efficiently taken up by both types of acceptor cells. Altogether, this suggests that extracellular circLSD1-RNAs can be taken up by cells of the environment. Interestingly, our findings point to a difference in release and uptake depending on the donor and acceptor cell type and provide primary insights into the divergent potential of cancer and non-cancer cells in circLSD1-RNA transfer.

## 3. Discussion

In our study, we identified four circRNA deriving from the exon 2 and exon 3 region of the *KDM1A* locus and named them accordingly: circ 2, circ 2a_2, circ 3_2, and circ 3_2_2a. FISH and a detailed quantitative analysis of the nuclear and the cytoplasmatic fractions showed that the majority of circLSD1-RNAs was in the cytoplasm. The location of a high proportion in the cytoplasm and minor enrichment in the nucleus is in agreement with recent observations of Zhang et al. [40]. Nuclear circRNAs might be involved in trancriptional regulation [41], whereas cytoplasmatic circRNAs can function, for example, in miRNA and protein sponging, or they might even be translated [20]. Thus, the enrichment of the circLSD1-RNAs in the cytoplasm points to a putative function in protein interactions, or in the RNA network as an RNA scaffold or vehicle, although the mechanistic links of the circLSD1-RNAs described here are not yet known. To date, there is only one report on this issue, in which Song et al. described a marked repression of circLSD1 3_2_2a (hsa_circ_0009061) in prostate cancer [32]. The in silico analysis predicted its binding to miR-103a-2-5p, miR-875-3p, miR-92-5p, miR-608, or miR-518c-5p. Importantly, in this study, it was also shown that circLSD1-3_2_2a is identified as one of the most dysregulated circRNAs in prostate cancer, which was strongly associated with the clinical outcome [32]. Consistently, when comparing the relative expression of circLSD1-RNAs to LSD1 mRNA between PC9 LUAD cells and PSAE non-cancer cells, we demonstrated that the expression is downregulated in the LUAD cells. Moreover, the quantitative analysis of clinical LUAD samples and the matching non-tumor tissues revealed a significant reduction of circLSD1 RNA levels in tumor specimens. The loss of the circLSD1-RNAs in the tumor samples let us assume that the circLSD1-RNAs were released from cancer cells after their dedifferentiation and transformation. Indeed, circRNAs are shown to be released into the blood stream from tumors and are considered to serve as non-invasive biomarkers, as previously shown for miRNAs [42,43]. Corresponding to the tumor-released miRNA, circulating extracellular circRNA was also found in extracellular vesicles of the blood and other body fluids [25]. Hence, hsa_circ_002178 was found with high sensitivity and specificity (AUC of 0.9956) in small EVs obtained from the plasma of patients with LUAD, indicating its potential to serve as a novel diagnostic biomarker for LUAD [44]. In our study, there were no serum samples of patients with LUAD available, but we detected two circLSD1-RNAs, circ 2 and circ 2a_2, in the serum of patients with T-PLL. Interestingly, these circLSD1-RNAs were only highly present in T-PLL samples, but not in serum samples of healthy controls, suggesting the release of these circLSD1-RNAs from tumor cells.

To monitor circLSD1-RNA release, we performed RNA extraction and qPCR on the cell culture media of PC9 and PSAE cells, which endogenously or transgenically expressed circLSD1-RNAs. The transgenic overexpression of circRNA was based on plasmids designed to promote circularization [45]. Corresponding to endogenous circLSD1-RNAs, which were released by both PC9 and PSAE cell types into the cell culture media, overexpressed circLSD1-RNAs and the control circGFP-RNAs were also efficiently delivered by both PC9 and PSAE cells.

We hypothesize that circLSD1-RNAs are packaged and released by the endo-/exocytic pathway, as was previously described for miRNAs [27]. Notably, we proved that extracellular circLSD1-RNAs released from PC9 cancer cells are protected by a membrane layer, confirming that they are enclosed in vesicles. Released EVs could be classified according to their size and the presence of specific membrane markers [46]. The EVs released from PC9 and PSAE cells had an average diameter of about 100 nm, which is in accordance with the size of exosomes/small EVs [38,39]. Furthermore, we show that isolated EVs from PC9 cells were positive for the presence of EV-associated proteins TSG101, CD63, and CD9. In contrast, EVs released by PSAE cells express significantly fewer of those markers, and, after circLSD1-RNA overexpression, these markers were completely lost, as shown by immunoblotting and proteomic profiling. This suggests a different sorting mechanism of not only circLSD1-RNA but generally for all RNAs/proteins released from PSAE cells.

The different pattern of EV markers in the EV fractions, obtained from supernatants of PC9 and PSAE cells, is in agreement with the different sensitivities to RNAse A degradation of circLSD1 RNAs derived from PC9 cancer cells versus PSAE non-cancer cells. Whereas circLSD1 RNAs, which we found in EV fractions from PC9 cells, were sensitive to RNase A digestion after Triton-X-100-mediated membrane solubilization, they were stable when originating from supernatants of PSAE cells. Therefore, we conclude that circLSD1-RNAs released by PSAE cells are additionally protected. A possibility is that circLSD1-RNAs from PSAE cells are associated with high- and low-density lipoproteins and could, therefore, be co-purified with EVs [47]. Furthermore, previous data have shown that circRNAs are also bound to miRNAs that, in turn, aggregate with RNA-binding proteins, such as AGO1 and AGO2 proteins [48]. Therefore, circLSD1-RNAs from PSAE might be additionally shielded from RNAse A degradation by their interaction with RNA-binding proteins and aggregation in RNP complexes. Indeed, proteomic analysis proved a great difference in the peptide profiles of EV-associated proteins obtained from PSAE non-cancer cells versus PC9 cancer cells. Importantly, miRNA-associated proteins and RNA-binding proteins such as the AGO1 and AGO2 proteins prominently differed in the EV peptide pattern. This indicates that the secretion process of extracellular circLSD1-RNAs underlies regulatory mechanisms which function in a cell-type-dependent manner, as already shown for miRNA sorting for export [49].

To study the uptake of circLSD1-RNA by PC9 and PSAE acceptor cells, we used conditioned media from both cell types, as well as purified small EVs or a mixture of small EVs with RNP complexes from PC9 or PSAE donors, respectively. The fate of RNA cargo delivered by EVs highly depends on EV internalization and the subsequent release of RNA content. RNA cargo may be released into the cytoplasm of the acceptor cells or remain in early endosomes until their maturation to late endosomes, from where they may be transported to lysosomes for degradation. The increase in circLSD1-RNAs as well as circGFP RNA in both acceptor cell types clearly indicates a general uptake process, although circ 2a_2 is most efficiently taken up by both acceptor cell types, pointing to sequence- or size-specific uptake into the cells. In addition, the intracellular fate and the function of the circRNAs uptaken into acceptor cells are suggested to be different and need to be investigated in future experiments.

Altogether, this study provides an insight into the role of circRNAs derived from the parental gene in the regulation of the expression of an important chromatin modulator protein, LSD1. This includes their participation in intercellular communication via EVs or RNPs, which potentially affects the acceptor cells. However, it is important to note that there are still several unanswered questions. Apart from investigating the circLSD1-RNA function, we want to address the fundamental question of how these circLSD1-RNAs are packaged into EVs and how this may affect their processing by acceptor cells. To provide us with more insight into the function of the identified circLSD1-RNAs, miRNA sequencing of the circLSD1-RNA containing EVs may also be performed to identify miRNA interactors. Due to the differences observed between cancer and non-cancer cells, it would be interesting to check for protein- and miRNA-binding partners that are involved in oncogenesis or tumor suppression. Moreover, with the increasing knowledge of the role of EVs in intercellular communication, our future studies on the participation of identified circLSD1-RNAs and their implications in the cancer-and-non-cancer-cell crosstalk will be of great interest.

## 4. Materials and Methods

### 4.1. Cell Culture

Human LUAD (PC9, ECACC 90071810 and A549, ECACC 86012804) and human primary small airway epithelial (PSAE, ATCC PCS-301-010) cells were used in this study. PC9 and A549 cells were cultured in Dulbecco’s Modified Eagle Medium (DMEM, Gibco, Thermofisher Scientific, Darmstadt, Germany) with 10% fetal bovine serum (FBS) while PSAE cells were cultured in Airway Epithelial Cell Basal Medium (ATCC, Virgin Islands, USA, https://www.atcc.org/, accessed on 9 September 2023) with the following supplements from the Bronchial Epithelial Cell Growth Kit (ATCC, Virgin Islands, USA): HLL supplement, L-glutamine, Extract P, and Airway Epithelial Cell Supplement. Cells were incubated at 37 °C with 5% CO_2_.

### 4.2. Cell Fractionation

Subcellular fractionation was performed according to the published protocol [50]. Briefly, PC9, A549, and PSAE cells were harvested by trypsinization, washed twice with cold PBS, and resuspended in 1 mL cold HMKE buffer (20 mM HEPES, pH 7.2, 10 mM KCl, 4 mM MgCl_2_M, 1 mM EDTA, 1 mM phenylmethylsulfonyl fluoride, 250 mM sucrose, protease inhibitor cocktail (Roche, Cologne, Germany), 200 µg/mL digitonin). After 10 min incubation on ice, nuclei were pelleted by centrifugation at 500× *g* at 4 °C for 10 min. The supernatant was collected as the cytoplasmic fraction. Nuclei were washed once with HMKE buffer, resuspended in 1:1 mixture of glycerol buffer (20 mM Tris-HCl pH 7.5, 75 mM NaCl, 0.5 mM EDTA, 0.85 mM DTT, 50% glycerol, protease inhibitor cocktail, 2 tablets) and nuclei lysis buffer (10 mM HEPES pH 7.5, 1 mM DTT, 7.5 mM MgCl_2_, 0.2 mM EDTA, 300 mM NaCl, 500 mM urea, 1% Triton X-100), and incubated on ice for 2 min. Chromatin was pelleted at 7000× *g*, 4 °C, for 2 min, and the supernatant, harboring the chromatin-depleted nucleoplasmic fraction, was cleared by centrifugation at 16,000× g, 4 °C for 2 min. The pellet with the chromatin fraction was resuspended in buffer C (20 mM HEPES pH 7.9, 1 mM DTT, 1.5 mM MgCl_2_, 150 mM NaCl, 50% Glycerol, 0.2 mM EDTA, 0.5 mM PMSF) and sonicated for 20 min using the Diagenode Bioruptor^®^ (Diagenode, Seraing, Belgium). After sonication, sheared chromatin was centrifuged at 16,000× *g*, 4 °C, for 5 min to remove the non-solubilized rests, and the supernatant, containing the solubilized chromatin, was collected for use in the downstream experiments.

### 4.3. Cloning of circLSD1-RNA for Overexpression

The vector for the overexpression of circGFP-RNA is based on the mammalian expression vector pIRESneo3 (https://www.addgene.org/vector-database/3183/, accessed on 9 September 2023), but the GFP sequence was added and flanked with inverted repetitive sequences from ZSCAN1 pre-mRNA [36] as shown in the Supplementary Information (Supplemental Figure S2). The vector was a kind gift from S. Bessonov (Institute for Epigenetics, University of Cologne, Cologne, Germany) and was used to insert the circLSD1-RNA-encoding sequences: LSD1-Exon2, LSD1-Exon2_2a, LSD1-Exon2_3, and LSD1-Exon2_2a_3. To this end, corresponding LSD1 exonic sequences were amplified from PC9 total cDNA, using first primer sets spanning exon 1 to exon 4 of LSD1 and then circRNA-specific primers (Supplementary Table S4) containing restriction enzyme sequences for PstI and NsiI in their tail sequences. Restriction enzyme digestion was then performed, and the resulting PCR products were inserted into the circGFP-RNA vector using T4 DNA ligation. The sequence of recombinant plasmids was confirmed by Sanger sequencing (Eurofins Genomics, Ebersberg, Germany).

### 4.4. Transgenic circLSD1-RNA Expression in PC9 and PSAE Cells

For the overexpression of circLSD1- and circGFP-RNAs, PC9 and PSAE cells were seeded in 12-well plates at 180,000 cells/well. The following day, transfection was performed using jetPRIME^®^ transfection reagent (PolyPlus) according to the manufacturer’s protocol. Then, 6 h after transfection, the cell media was changed to get rid of the rest of the transfection reagent and plasmids. The next day, 24 h after, transfection cells were harvested. Cell culture media were cleared by centrifugation at 2500 rpm for 20 min at RT and used for the downstream applications.

### 4.5. Treatment of PC9 and PSAE Cells with circLSD1-RNA Conditioned Media

PC9 and PSAE acceptor cells were seeded in 12-well plates and, the following day, cell culture media were replaced by media, which were collected from PC9 and PSAE donor cells 24 h after their transfection with circGFP or circLSD1-Exon2, circLSD1-Exon2a_2, circLSD1-Exon3_2, and circLSD1-Exon3_2_2a vectors for the overexpression. The acceptor cells were then incubated for 24 h and harvested for the downstream experiments.

### 4.6. Fluorescence In Situ Hybridization (FISH)

Fluorescence in situ hybridization was performed in PC9 cells using antisense LNA/DNA probes (QIAGEN, Hilden, Germany) complementary to the back-splicing junction of circ 2a_2 and circ 3_2 LSD1-RNA according to the published protocol [51]. U6 snRNA DNA antisense probe (Eurofins Genomics, Ebersberg, Germany) was used in the control setups. Probes were fluorescently tagged with Cy3, and their sequences are listed in Supplementary Table S2.

### 4.7. Extracellular Vesicle Isolation

Extracellular vesicles were isolated from PC9 and PSAE cell culture media by the precipitation method. First, the conditioned media were collected from cell cultures, centrifuged at 2000× *g* for 30 min to remove cell debris, and then used for extracellular vesicle (EV) purification using the “Total Exosome Isolation Reagent” (Invitrogen, Life Technologies, Darmstadt, Germany) following the manufacturer’s instructions. Briefly, the TEI reagent and cell culture medium were mixed in a 2 mL tube with a ratio of 1:2 and samples were incubated at 4 °C overnight. The next day, samples were centrifuged at 10,000× *g*, 4 °C, for 1 h. The supernatant was discarded and the precipitated EVs were resuspended in 10 µL of Dulbecco’s PBS buffer.

### 4.8. Nanoparticle Tracking Analysis

To determine the size distribution of the EVs, we performed nanoparticle tracking analysis. Isolated EVs were first bulked to 50 µL with DPBS, then diluted further at 1:100 with DPBS, before being injected into the ZetaView^®^ Nanoparticle Tracking Analyzer (Analytik Ltd., Cambridge, UK) as previously described [52].

### 4.9. Treatment of PC9 and PSAE Cells with Purified EVs

PC9 and PSAE acceptor cells were seeded in 12-well plates at 1,800,000 cells per plate. Next day, cell culture media were replaced by fresh media, containing EVs isolated from PC9 and PSAE donor cells 24 h after their transfection with circGFP or circLSD1-Exon2, circLSD1-Exon2a_2, circLSD1-Exon3_2, and circLSD1-Exon3_2_2a vectors for the overexpression of circRNAs. EVs were resuspended in Dulbecco’s PBS buffer and filtered using 0.45 µm filter before being distributed to PC9 and PSAE acceptor cells. The acceptor cells were then incubated for 24 h and harvested for the downstream experiments.

### 4.10. Western Blot Analysis

Samples were reduced by addition of Laemmli buffer with 0.7 M β-mercaptoethanol and boiled at 65 °C for 10 min. Protein samples were then loaded and run in 12% SDS-PAGE gels, at 100 V until the end of the stacking gel and at 130 V in the separating gel, after which membrane protein transfer on methanol-activated 0.2 µm PVDF membrane was performed using the Mini Trans-Blot^®^ Cell (BioRad, Duesseldorf, Germany) at 90 V for 2 h. Blocking of the membrane with 5% (*w/v*) milk in PBS-T buffer, containing 0.05% Tween 20, was carried out for 1 h, followed by incubation with the primary antibody in 5% (*w/v*) milk/PBS-T overnight at 4 °C. Primary antibodies which we used are indicated in Supplementary Table S3. The following day, the membrane was washed with 0.05% PBS-T, then incubated with the corresponding secondary antibodies, listed in Supplementary Table S3, in 5% (*w/v*) milk/PBS-T at RT for 1 h. After incubation, the membranes were washed with PBS-T, and Pierce^TM^ ECL Western Blotting Substrate (ThermoFisher Scientific, Darmstadt, Germany) was added for chemiluminescence. Imaging was then performed on the BioRad ChemiDoc^TM^ Imager (BioRad, Munich, Germany).

### 4.11. RNA Extraction

Isolation of RNA from cells, lung tissues, cell culture medium, serum, or purified extracellular vesicles was performed with TRIzol^®^ (Ambion, Life Technologies, Darmstadt, Germany) according to the manufacturer’s protocol.

### 4.12. Reverse Transcription, and Qualitative and Quantitative PCR

cDNA synthesis was performed using SuperScript™ III Reverse Transcriptase (Invitrogen, Life Technologies, Darmstadt, Germany) following the manufacturer’s protocol. GoTaq^®^ qPCR Master Mix (Promega, Wittmund, Germany) was used to perform real-time PCR. All assays were carried out in technical triplicates. Relative expression was calculated by the ΔΔCt method using HPRT or GAPDH as housekeeping genes.

Qualitative PCR was performed with of GoTaq^®^ Hot Start Master Mix (Promega, Wittmund, Germany). Samples were then loaded in a 1.8% agarose gel containing GelRed^®^ (Biotium, Fremont, CA, USA) and run at 100 V. Gels were visualized using the Bio-Rad ChemiDoc™ Imager. Primers used are listed in Supplementary Table S1.

### 4.13. Statistical Analysis

Experiments were performed in triplicates, unless otherwise indicated in the figure legend. Comparisons between two groups were performed by Student’s *t*-test, and, when more than two groups were analyzed, we used the one-way ANOVA and Tukey post-hoc tests. Graphs were prepared using the GraphPrism software V5 and values were presented as mean ± standard deviation. Data were considered as significant when the *p*-values are <0.05, and the *p*-values were indicated as follows: * *p* < 0.05; ** *p* < 0.01; *** *p* < 0.001.

### 4.14. Proteomic Analysis by Mass Spectrometry 

The purified extracellular vesicles with an average protein concentration of 300–500 µg/mL in 20 µL were processed according to CECAD/CMMC, University of Cologne, Proteomics Core Facility protocol (https://www.cecad.uni-koeln.de/research/core-facilities/proteomics-facility, accessed on 5 June 2023). Briefly, proteins from each sample were reduced with DTT, then alkylated by using chloroacetic acid (CAA). The samples were digested for 4 h with endoproteinase *Lys-C*, then overnight with trypsin, followed by acidification with formic acid to stop enzymatic digestion. The resulting peptides were purified by using stage tips containing C18 resin and submitted to the Proteomics Facility of the CECAD (CECAD—Cluster of Excellence, University of Cologne, Germany) for global proteome quantification.

The mass spectrometry data were analyzed using the Perseus computational platform [53]. For whole proteome analysis, only ANOVA-significant protein was used, and a threshold of *p* < 0.05 was used. For interactome analysis, Student’s *t*-test *p*-values were used (*p* < 0.01). Gene ontology analysis was performed using the Instant Clue software [54].

## Figures and Tables

**Table 1:** Characteristics of circRNAs, deriving from the exon 2-2a-3 locus of the *KDM1A* gene.

Identified crcRNA	Locus	CircRNA Accession No *	Spliced Length	LSD1 Exons
circ-3_2_2a_LSD1-RNA	chr1:23356961–23377013	hsa_circ_0009061	360 b	2, 2a, 3
circ-3_2_LSD1-RNA	chr1:23356961–23377013		300 b	2, 3
circ-2a_2_LSD1-RNA	chr1:23356961–23370979	hsa_circ_0112434	226 b	2, 2a
circ-2_LSD1-RNA	chr1:23356961–23357127		166 b	2

b: bases; * accession number from the circRNA database (circBase).

**Table 2:** Pathways divergently involved in circRNA release by PC9 versus PSAE cells.

Pathway	*p*-Value	FDR
Deadenylation-dependent mRNA decay	1.11 × 10^−16^	5.01 × 10^−14^
mRNA decay by 5′ to 3′ and 3′ to 5′ exoribonuclease	6.57 × 10^−10^	1.48 × 10^−7^
Metabolism of RNA	5.34 × 10^−8^	5.66 × 10^−6^
Glycolysis, glucose, and carbohydrate metabolism	6.28 × 10^−8^	5.66 × 10^−6^
Deadenylation of mRNA	1.04 × 10^−6^	6.67 × 10^−5^
mRNA splicing	9.14 × 10^−3^	0.338
Major pathway of rRNA processing in the nucleolus and cytosol	1.95 × 10^−2^	0.472

## Supplementary Materials

The following supporting information can be downloaded at: https://www.mdpi.com/article/10.3390/ijms241813981/s1.
