# Supplementary material for: Vesicular Release and Uptake of Circular LSD1-RNAs from Non-Cancer and Cancer Lung Cells
Source: Int J Mol Sci. 2023 Sep 12;24(18):13981. doi: 10.3390/ijms241813981 (PMC10530930; doi:10.3390/ijms241813981)
Supplement: Supplementary file 1 [file ijms-24-13981-s001.zip › ijms-2547409-supplementary.pdf]

Supplementary Information

Vesicular Release and Uptake of Circular LSD1-RNAs from Non-Cancer and Cancer Lung Cells

Joelle Noriko Galang [1,2,†,‡], Yefeng Shen [1,2,†,§], Ulrike Koitzsch [1,2], Xiaojie Yu [1,2], Hannah Eischeid-Scholz [1,2], Daniel Bachurski [3,4], Tilman T. Rau [5], Christina Neppl [5], Marco Herling [4,6], Bianca Bulimaga [1,2], Elena Vasyutina [4,6], Michal R. Schweiger [2,7], Reinhard Büttner [1,2], Margarete Odenthal [1,2,*] and Maria M. Anokhina [1,2,5,*]

Supplementary Figures S1–S5

Supplementary Tables S1–S4

Supplemental Figures

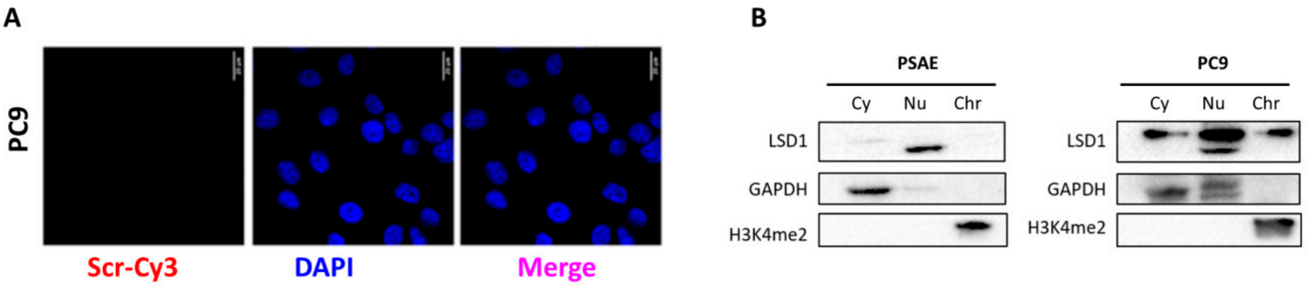

Supplementary Figure S1. (A) Fluorescence in situ hybridisation (FISH) on PC9 cells using a scramble probe, fluorescently tagged with Cy3. (B) Cellular fractionation was performed on PSAE and PC9 cells to obtain the cytoplasmic, nucleoplasmic, and chromatin fraction. Western blot of PSAE and PC9 cellular fractions is shown. Antibodies recognizing LSD1 as a nuclear fraction marker, GAPDH as a cytoplasmic fraction marker, or H3K4me2 as a chromatin fraction marker were used.

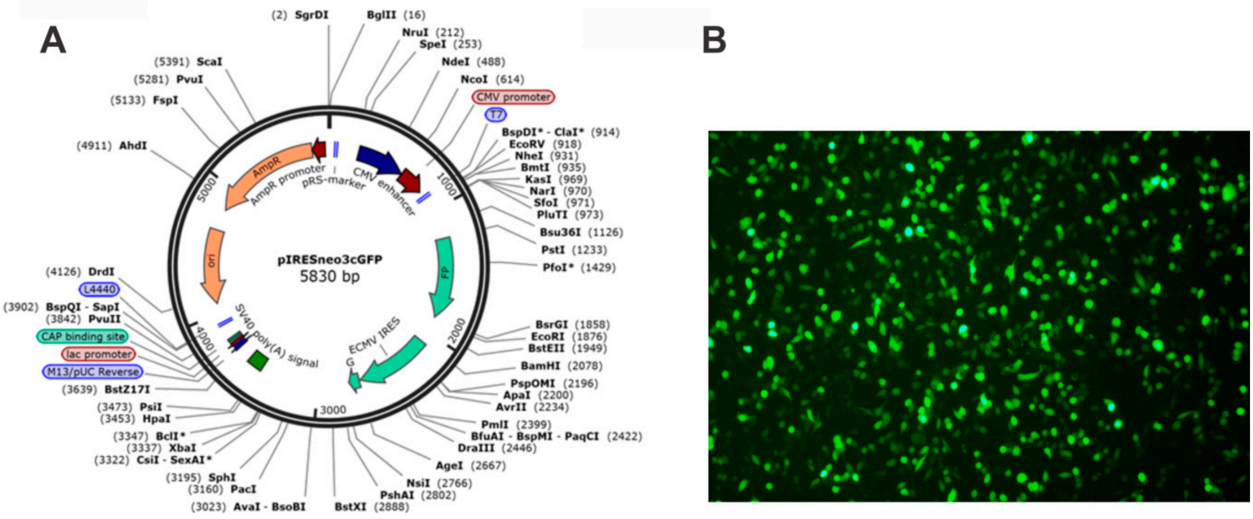

Supplementary Figure S2. Transgenic circGFP-RNA expression

(A) Plasmid map of pIRESneo3 cGFP plasmid , used as a reporter control for expression and forming of circRNA. (B) GFP expression in PC9 cells, transduced with the pIRES neo3GFP plasmid shown in (A).

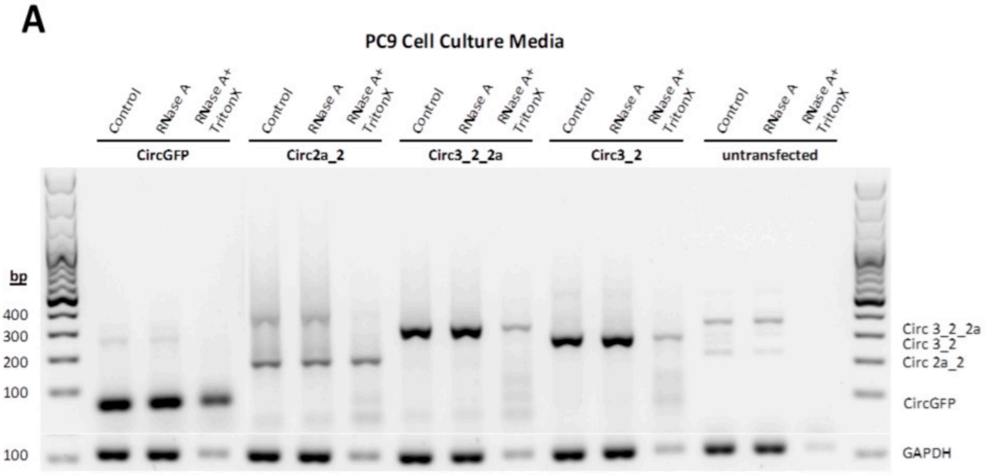

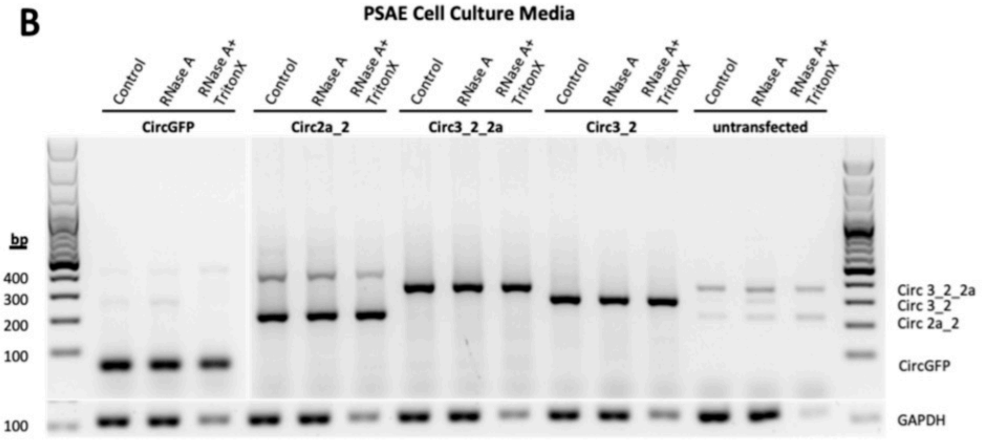

Supplementary Figure S3. Protection of circLSD1 RNA from RNAseA degradation

PC9 (A) and PSAE cells (B) were transfected with plasmids expressing CircGFP and the circLSD1 RNAs Circ2a-2, Circ3-2-2a, Circ3-2 and the cell supernatants were collected. RNA was then extracted from PC9 (A) and PSAE cell culture media (B) after RNase A or the combined RNase A plus Triton X-100 treatment. Agarose gel images after reverse transcription and PCR amplification of circLSD1-RNA using divergent primers are shown.

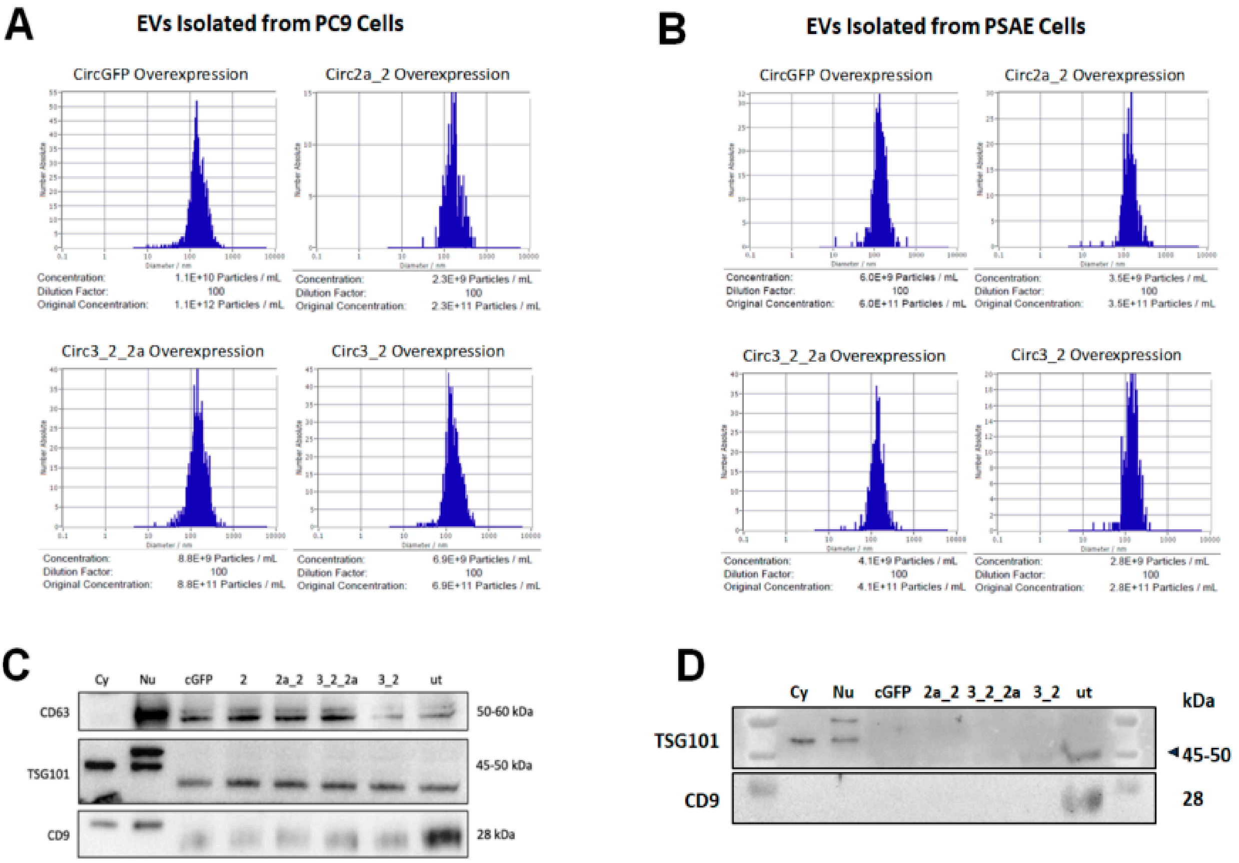

Supplementary Figure S4. Characteristics of EV released from PC9 cancer and PSAE non-cancer cells

(A,B) Size distribution graphs of isolated EVs from PC9 (A) and PSAE cells (B) after transgenic overexpression of the circLSD1-RNAs Circ2a-2, Circ3-2-2a, Circ3-2 or the circGFP (cGFP), which was used as a control. (C, D) Immunoblotting for EV markers CD63 (C), TSG101 (C,D), and CD9 (C,D) in isolated EVs from PC9 cells after circLSD1-RNA overexpression (ut: untransfected).

A **mRNA decay, RNA stress and sensing pathway**

Cluster ID	PC9 LUNG CANCER			PSAE NON-CANCER			Protein	First.Protein.Description
Cluster (2)	-2.04	-1.81	-1.82	1.22	1.12	0.72	LSM2	U6 snRNA-associated Sm-like protein LSm2
	-2.06	-1.87	-1.88	1.29	0.78	0.80	AGO1	Protein argonaute-1
	-2.06	-1.55	-1.58	0.91	0.68	0.30	LSM8	U6 snRNA-associated Sm-like protein LSm8
	-1.50	-1.36	-1.37	1.07	0.86	0.88	LSM4	U6 snRNA-associated Sm-like protein LSm4
	-1.45	-1.21	-1.22	1.12	1.39	1.38	LSM3	U6 snRNA-associated Sm-like protein LSm3
	-1.24	-1.07	-1.08	1.40	-0.33	1.14	LSM6	U6 snRNA-associated Sm-like protein LSm6
	-2.11	-2.43	-2.40	1.30	1.42	0.48	TSN	Translin
	-1.54	-2.66	-2.68	1.46	0.56	0.56	AGO2	Protein argonaute-2
	-1.75	-2.09	-2.89	1.15	-0.09	-0.13	DDX6	Probable ATP-dependent RNA helicase DDX6
Cluster (1)	-0.67	-0.77	-1.21	-0.36	-1.40	-1.29	DDX5	Probable ATP-dependent RNA helicase DDX5
	-0.29	-0.87	-2.19	0.46	-0.69	-0.94	PRKRA	Inf-inducible dsRNA-proteinase kinase act A
	0.37	0.06	-1.62	-0.40	-0.51	-0.70	EDC4	Enhancer of mRNA-decapping protein 4

B **hnRNP proteins**

Cluster ID	PC9 LUNG CANCER			PSAE NON-CANCER			Protein	First.Protein.Description
Cluster (2)	-0.46	-1.01	-0.82	-2.07	-2.21	-0.87	SYNCRIP	HN ribonucleoprotein Q
	0.31	-0.15	-0.09	-0.73	-1.47	-1.29	HNRNPC	HN ribonucleoproteins C1/C2
	-0.04	-0.87	-0.50	0.25	-3.56	-0.62	HNRNPF	HN ribonucleoprotein F
	0.32	-1.45	-1.44	-0.33	-0.43	-0.60	HNRNPH3	HN ribonucleoprotein H3
	0.29	-1.60	0.03	-0.41	-0.52	-0.70	HNRNPUL1	HN ribonucleoprotein U-like protein 1
	-0.06	-2.27	-0.21	0.55	-0.76	-1.01	HNRNPA0	HN ribonucleoprotein A0
	-0.21	-2.36	-0.20	0.60	-1.52	-1.66	HNRNPDL	HN ribonucleoprotein D-like
	-0.16	-1.26	-0.86	0.37	-1.47	-1.37	HNRNPA2B1	HN ribonucleoproteins A2/B1
Cluster (1)	-1.77	-2.71	-1.94	0.84	-0.77	-0.47	HNRNPUL2	HN ribonucleoprotein U-like protein 2
	-1.74	-2.56	-1.97	0.65	-1.09	-0.26	HNRNPR	HN ribonucleoprotein R
	-1.69	-2.62	-2.12	1.47	-1.21	0.01	HNRNPL	HN ribonucleoprotein L
	-1.02	-2.55	-1.73	0.58	-1.33	-0.80	HNRNPM	HN ribonucleoprotein M
	-1.46	-2.57	-1.67	0.80	-0.43	-0.80	HNRNPU	HN ribonucleoprotein U
	-1.80	-2.17	-2.25	1.18	-0.02	-0.69	HNRNPAB	HN ribonucleoprotein A/B
	-1.96	-2.48	-2.05	1.60	0.35	0.32	HNRNPD	HN ribonucleoprotein D0
	-1.66	-2.78	-2.40	1.63	0.42	0.41	HNRNPK	HN ribonucleoprotein K
	-1.60	-2.84	-2.32	1.74	-0.07	0.05	HNRNPA3	HN ribonucleoprotein A3

HN: Heterogeneous nuclear

Supplementary Figure S5: Protein pattern of EV fractions obtained from PC9 and PSAE cells

Different pattern of proteins involved in the mRNA decay and RNA stress pathways (A) as well as a different profile of HNRNP proteins (B) in EV fractions, that were obtained from PSAE and PC9 cells. The fold change values (FC) in EV fractions obtained from PC9 versus PSAE cells are indicated.

Supplemental Tables

Supplementary Table S1. List of primers used for quantitative and qualitative PCR

Primer name	Orientation	Sequence (5'-3')	Primer description
HPRT gene	Forward	GACCAGTCAACAGGGGACAT	Convergent
HPRT gene	Reverse	GTGTCAATTATATCTTCCACAATCAAG	Convergent
Exon 1 of LSD1 gene	Forward	GAAACTGGAATAGCAGAGACTCC	Convergent
Exon 2 of LSD1 gene	Reverse	TTCTTCCTCAGGTGGGGCTT	Convergent/divergent
Exon 2a of LSD1 gene	Reverse	CGTCTCCATACCCTCCAGAA	Convergent
Exon 2 of LSD1 gene	Forward	AGTGAGCCTGAAGAACCATC	Divergent
Exon 3 of LSD1 gene	Reverse	ACCTTCTGGGTCTGTTGTGG	Convergent
Back-splicing junction of Exon 2_2 circRNA of LSD1 gene	Forward	ACCATCGGGTAGAGTACAGAGA	Convergent
Back-splicing junction of Exon 2a_2 circRNA of LSD1 gene	Forward	CAAGCATCAGGTAGAGTACAG	Convergent
Back-splicing junction of Exon 3_2 circRNA of LSD1 gene	Forward	GAAACCGCACAGTAGAGTACAG	Convergent
GAPDH gene	Forward	CATGAGAAGTATGACAACAGCCT	Convergent
GAPDH gene	Reverse	AGTCCTTCCACGATACCAAAGT	Convergent
GFP translated sequence	Forward	ACGTAAACGGCCACAAGTTC	Divergent
GFP translated sequence	Reverse	GTCAGCTTGCCGTAGGTGG	Divergent

Supplementary Table S2. FISH antisense oligonucleotide probe sequences

FISH probe	Sequence (5' – 3')
U6	TTTGCGTGTCATCCTTGCG
circ 2a_2 LSD1-RNA	GCCGGTTCGTAGTCGTAGA
circ 3_2 LSD1-RNAs	CTTTGGCGTGTCATCTCAT

Supplementary Table S3. Primary and secondary antibodies used in this study

Antibody	Catalog Number	Host
Anti-CD63	10628D (Invitrogen)	Mouse
Anti-CD9	EXOAB-KIT-1-SBI (SBI)	Rabbit
Anti-TSG101	Ab125011 (Abcam)	Rabbit
Secondary Anti-mouse	Ab6728 (Abcam)	Rabbit
Secondary Anti-rabbit	Ab6721 (Abcam)	Goat
GAPDH	Ab9485 (Abcam)	Goat
H3K4me2	9725 Cell (Signaling	Rabbit
LSD1	Ab37165 (Abcam)	Rabbit

Supplementary Table S4. Primers used for cloning of circLSD1-RNA

Primer Name/Description	Orientation	Sequence (5'-3')
Exon1 of LSD1 gene	Forward	GAAACTGGAATAGCAGAGACTCC
Exon4 of LSD1 gene	Reverse	TGTTGGAGAGTAGCCTCAAATGTC
Exon2_PstI	Forward	TATACTGCAGGTAGAGTACAGAGAGATGGATGAAAGC
Exon3_ NsiI	Reverse	AATTATGCATACTCACTGTGCGGTTTCTAATGAAAAGAAAAAC
Exon2a_ NsiI	Reverse	AATTATGCATACTCACCTGATGCTTGGCCGTCTCCA
Exon2_ NsiIe	Reverse	AATTATGCATACTCACCCGATGGTTCTTCAGGCTCACTT
